# Can the current monkeypox affect the heart? A systematic review of case series and case report

**DOI:** 10.1186/s12872-023-03351-3

**Published:** 2023-06-28

**Authors:** Reem Sayad, Abdelmonem Siddiq, Ahmed Hashim, Ahmed Saad Elsaeidy

**Affiliations:** 1 Faculty of Medicine, Assiut University, Assiut, Egypt; 2 Faculty of Pharmacy, Mansoura University, Mansoura, Egypt; 3 Faculty of Medicine, Ain Shams University, Cairo, Egypt; 4 Faculty of Medicine, Benha University, Benha, Egypt

**Keywords:** Monkeypox, Mpox, Cardiomyopathy, Myocarditis, Carditis, Pericarditis

## Abstract

**Background:**

Monkeypox is a zoonotic viral infection first reported in May 2022. Monkeypox cases present with prodromal symptoms, rash, and/or systemic complications. This study systematically reviews the monkeypox cases presented with any cardiac complications.

**Methods:**

A systematic literature search was done to locate papers that discuss any cardiac complications associated with monkeypox; then, data were analyzed qualitatively.

**Results:**

Nine articles, including the 13 cases that reported cardiac complications of the disease, were included in the review. Five cases previously had sex with men, and two cases had unprotected intercourse, which reveals the importance of the sexual route in disease transmission. All cases have a wide spectrum of cardiac complications, such as acute myocarditis, pericarditis, pericardial effusion, and myopericarditis.

**Conclusion:**

This study clarifies the potential for cardiac complications in monkeypox cases and provides avenues for future research to determine the underlying mechanism. Also, we found that the cases with pericarditis were treated with colchicine, and those with myocarditis were treated with supportive care or cardioprotective treatment (Bisoprolol and Ramipril). Furthermore, Tecovirimat is used as an antiviral drug for 14 days.

## Introduction

Monkeypox (Mpox) is a viral zoonotic disease caused by the Mpox virus, a member of the *Orthopoxviral* genus from the *Poxviridae* family of viruses [1]. The first human Mpox case was detected in 1970 [2]. Mpox was mainly endemic to central and western Africa, with a few sporadic cases outside Africa. Traveling to the endemic regions and importing animals such as Squirrels and rodents from Ghana in 2003 led to the Mpox outbreak in the United States [3].

The first human case of Mpox-outbreak-2022 was reported to the WHO on May 07. In 2022 from a traveler who returned from Nigeria then, the cases started to increase, which prompted the WHO to declare the Mpox disease as a public health emergency of international concern to raise awareness about it and promote the countries' preparedness [4]. According to the Center for Disease Control and Prevention (CDC), the total number of Mpox cases is 87,078 in both the endemic and non-endemic sites of the disease [5].

The disease manifestation starts with a prodromal phase of fever, headache, myalgia, and lymphadenopathy, followed by the eruption of multiple swollen and umbilicated cutaneous lesions on the patient's face, hands, and genitalia. Anal pain and the other features last five days, a common period of *Orthopoxviruses*, followed by a rash. The rash proceeds over different phases, starting with macular, papular, vesicular, pustular, and finally, the crustation phase lasting from two to four weeks on common sites on the body such as the face, palm, and soles, and oral membranes. Certain complications may occur, such as cellulitis, encephalitis, corneal infection, and sepsis [6]. The above data points clearly to the diversity of the disease manifestations, but other manifestations are unknown till now, so we aim here to summarize the cardiac complications of the Mpox infection.

## Methods

We followed the approaches for conducting the current study based on the Cochrane Handbook of systematic reviews on Interventions [7]. While drafting our manuscript, we strictly followed the recommended reporting items for the Preferred Reporting Items for Systematic Reviews and Meta-Analyses (PRISMA) statement guidelines [8].

### Search strategy

Firstly, the following electronic databases were systematically searched: PubMed, Cochrane, Medline, Scopus, and Web of Science until December 01, 2022. We used the following search strategy in the previously mentioned databases: ((Monkeypox) AND (cardi* OR Heart OR myocarditis or cardiomyopathy)). All the included studies’ references were screened to avoid missing any studies and guarantee high-quality screening. Then, we updated the search process on April 29, 2023, during the revision round.

### Eligibility criteria

We included any primary study (case reports, case series, cohort) with patients suffering from heart injuries after the onset of Mpox symptoms. On the other hand, we excluded non-human studies, conference abstracts, reviews, and non-English studies.

### Screening and study selection

Using Endnote software (version X20.4.1), we collected the different records from the different databases and removed duplicates. The retrieved references were examined. The screening was done in two steps; title and abstract screening, followed by full-text screening for final eligibility. Two independent authors conducted the screening, and after comparing their findings, group discussions were used to settle any differences.

### Data extraction

The following data were extracted from the included studies by two independent authors. Study ID, Country, Study design, Age, Male gender, Presenting symptoms (Systemic, Non-systemic, and Cardiac), History of vaccinations, History of other etiology of myocarditis or infections, Homosexual/bisexual/MSM, Route of transmission, Immunocompromised, Treatment (Mpox and Myocarditis), Duration of hospitalization, and Investigation (Mpox, Myocarditis, and Follow-up). Arguments were later resolved through group discussion.

### Quality assessment

The quality of included studies was assessed using the National Institute of Health (NIH) quality assessment tool for case reports and case series. The tool assesses the quality of each study at the level of nine domains. Each domain, as well as the overall quality, is rated either good, fair, or poor [9]. Two independent authors assessed the quality of the included studies, and arguments were later resolved through group discussion.

### Data synthesis

Qualitative analysis was done by collecting and summarizing non-numerical data to understand symptoms, criteria, and how to manage the cases of Mpox associated with cardiac complications.

## Results

### Search results

**Fig. 1:**
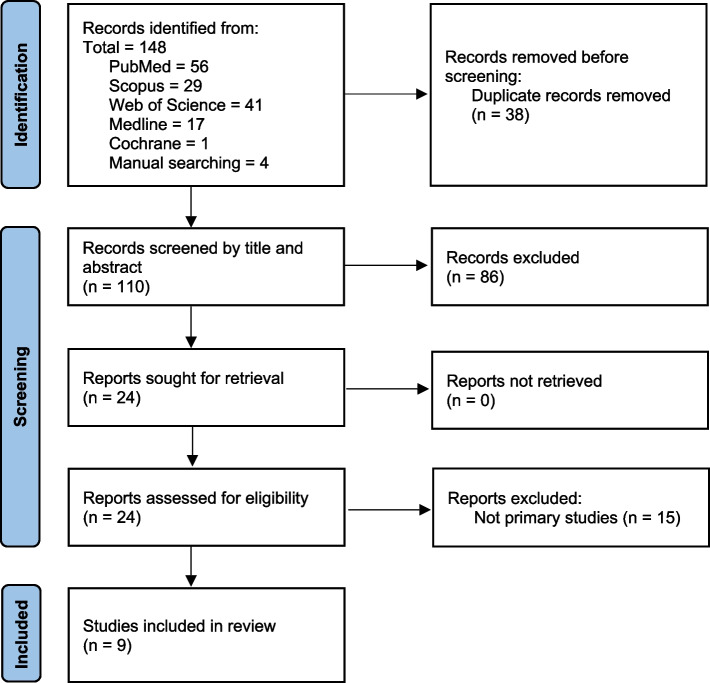
PRISMA flowchart of the database search and searching process

The search strategy over the different medical databases (PubMed, Scopus, Web of Science, Medline, Cochrane) yielded 144 studies. Four studies were retrieved by manual searching, so the total number of studies became 110 after removing duplication. Screening the title and the abstract yielded 24 studies after excluding eighty-six papers because of being animal studies, out of the study criteria, and/or not primary studies. Twenty-four articles were screened for their full-text testing for eligibility. Nine case reports/case series met the study criteria [3, 10–17] and were further included in the quality assessment, as shown in Fig. 1. A summary of the included papers can be found in Table 1.

**Table 1:** A summary of included studies

Study ID		Country	Study design	Age (Years)	Male gender	Presenting symptoms			History of vaccinations	History of other etiology of myocarditis or infections	Homosexual /bisexual/MSM
						**Systemic**	**Non-systemic**	**Cardiac**			
Santaliz-Ruiz et al. 2023 [17]		Puerto Rico	Case Report	21	Yes	Fever, Myalgia, Nausea, Vomiting, Headache, and Watery Diarrhea, Cervical Lymphadenopathy	Vesiculopustules on face, trunk, suprapubic area, and Extremities	Retrosternal non-radiating oppressive chest pain	NO	No	Yes
Brouillard et al. 2022 [15]		Canada	Case Report	34	Yes	Fever, Chills	A well-circumscribed, umbilicated papule on the pubis, and an ulcerated lesion on the glans penis	Constant, sharp, and pleuritic non radiating chest pain, relieved when sitting upright and worse when lying down	No	No, but later presented positive for Chlamydia and negative for Gonorrhea	NO, but stayed at a homosexual friend’s house two weeks earlier
Dumont et al. 2022 [14]	Case 1	France	Case series	21	Yes	Fever	Anal pustules	Acute chest pain radiating into the arms and jaw appeared	No	No	Yes
	Case 2			25	Yes	NA	Pustules on the face and penis	Constant chest pain and palpitations	No	No	NA
	Case 3			32	Yes	Fever	Erosive cutaneous lesions on the penis	Retrosternal chest pain radiating to the left arm	No	Slightly detectable Epstein-Barr virus DNA in blood < 500copies/mL	No
Miller et al. 2022 [16]		USA	Case-series	In his 302	Yes	NA	Rash on his face, head, back, and genitals then spread, coalesced, and developed central necrosis, Phimosis, urinary retention	Atrial Fibrillation	NA	HIV, Syphilis	NA
Pinho et al. 2022 [10]		Portugal	Case Report	31	Yes	Fever, Malaise, Myalgia	Eruption of multiple swollen and umbilicated cutaneous lesions on his face, hands, and genitalia	Chest tightness radiating to the left upper extremity that awoke him during the night	Prophylaxis against HIV	NO, Paucisymptomatic SARS-CoV-2 infection 2 months before this event	Yes
Rodriguez-Nava et al. 2022 [11]	Case 1	USA	Case-series	32	Yes	Viral illness, Cervical Lymphadenopathy	Disseminated rash and a painful penile lesion	Chest pain and dyspnea for 1 day	No	Syphilis treated by doxycycline	Yes
	Case 2			37	Yes	Fever, Dyspnea, Inguinal Lymphadenopathy, Decreased Exercise Tolerance	Multiple skin lesions in both arms and a lesion at the base of the penis	Difficulty breathing and decreased exercise tolerance without chest pain	Prophylaxis against HIV	Syphilis	NA
Shaik et al. 2022 [12]		USA	Case-Report	51	Yes	Fever, Fatigued, Flu-like symptoms, Malaise	Developed several vesiculopustular lesions on his face and extremities	Retrosternal chest pain radiating to the left arm	NA	NA	NA
Tan et al. 2022 [13]		Canada	Case-Report	40	Yes	Fever, Chills, Myalgia, headache	Umbilicated maculopapular and vesicular skin lesions over his chest, arms, Libs and genitals	Central, non-radiating, pressure-like chest pain but denied cough, palpitations, or shortness of breath	NO	Stable HIV (CD4 count = 609 cells/mm3, viral load undetectable, antiretroviral therapy)	Yes
Thornhill et al. 2022 [3]	Case 1	NA	Case-series	NA	NA	NA	NA	NA	NA	NA	NA
	Case 2			NA	NA	NA	NA	NA	NA	NA	NA

Study ID		Route of transmission	Immunocompromised	Treatment		Duration of hospitalization	Investigation		
				**Mpox**	**Myocarditis**		**Mpox**	**Myocarditis**	**Follow-up**
Santaliz-Ruiz et al. 2023 [17]		Sexual transmission	No	NA	High-dose Aspirin, Oral Colchicine 0.6 mg daily	Five days	PCR assay of a swab sample from a skin lesion	CBC, CMP, ECG, Echo, Troponin, Cardiac MRI	Cardiac MRI after Eight weeks
Brouillard et al. 2022 [15]		Contact with soiled linens of a homosexual friend	No	- Prophylactic Ceftriaxone and Azithromycin - Acyclovir in negative pressure room - Tecovirimat for 14 day after confirmed diagnosis	ACE inhibitors	Nine days	PCR assay of a swab sample from a skin lesion	ECG, Echo, Cardiac MRI, Troponin, Standard labs	The patient left the hospital before undergoing his control cardiac MRI
Dumont et al. 2022 [14]	Case 1	Sexual transmission	No	NA	Bisoprolol and Ramipril	NA	PCR assay of a swab sample from a 14skin lesion	ECG, Troponin, CK, TTE, CRP, contrast CT chest	Cardiac MRI after four weeks
	Case 2	Sexual transmission	No	NA	Bisoprolol and Ramipril	NA	PCR assay of a swab sample from a skin lesion	ECG, Troponin, CK, CRP, TTE	NA
	Case 3	Sexual transmission	No	Oral Tecovirimat for 14 day after confirmed diagnosis	Bisoprolol, Ramipril and Anti-aggregation	NA	PCR assay of a swab sample from a skin lesion	ECG, Troponin, CK, TTE, CRP, Cardiac-CT Scan	Cardiac MRI after 11 days
Miller et al. 2022 [16]		NA	Yes	- Oral Tecovirimat for 14 day after confirmed diagnosis, then IV in ICU - 2 doses of VIGIV - Antimicrobials	NA	- 4 weeks in the 1^st^ admission - 15-day in the 2nd admission	PCR assay of a swab sample from a skin lesion	NA	NA
Pinho et al. 2022 [10]		NA	NA	NA	Supportive care and exercise restriction	One week	PCR assay of a swab sample from a skin lesion	ECG, Chest X-ray, TTE, CRP, CK, Troponin, Brain natriuretic peptide, urine and blood toxicology tests, Cardiac MRI	NO
Rodriguez-Nava et al. 2022 [11]	Case 1	Sexual transmission	No	Oral Tecovirimat for 14 day after confirmed diagnosis	No specific treatment due to the rapid resolution	10 days	PCR assay of a swab sample from a skin lesion	ECG, Troponin, Chest X-ray, CRP, ESR, N-terminal prohormone B-type Natriuretic Peptide	Echo on hospital day2, Troponin in hospital day 6
	Case 2	Sexual transmission	No	Supportive care	Supportive care	4 days	PCR assay of a swab sample from a skin lesion	ECG, Troponin, Echo, B-type natriuretic peptide	Cardiac enzymes testing
Shaik et al. 2022 [12]		NA	No	Supportive care	Nitroglycerin at presentation, 1 g aspirin/h for 2 weeks	7 days	PCR assay of a swab sample from a skin lesion	ECG,TEE, Chest X-ray, Troponin, CBC, ALT, AST, BUN, Cr, CRP, ESR, HbA1c	High-dose aspirin, and a planned cardiac follow-up
Tan et al. 2022 [13]		Sexual transmission	No	Valacyclovir for initial diagnosis consideration of HSV 1,or 2	Supportive care	NA	PCR assay of a swab sample from a skin lesion	ECG, CK, Echo, Cardiac Catheterization, Cardiac MRI, Troponin, Routine Chemistries, CBC	Full recovery after 25 days
Thornhill et al. 2022 [3]	Case 1	NA	NA	NA	self-limiting (< 7 days)	NA	NA	NA	NA
	Case 2	NA	NA	NA		NA	NA	NA	NA

*ALT* Alanine transaminase, *AST* Aspartate transaminase, *BUN* Blood urea nitrogen, *CBC* Complete blood count, *CK* Creatine Kinase, *CMP* Comprehensive Metabolic Panel, *Cr* Creatinine level, *CRP* C-reactive protein, *ECG* Electrocardiography, *Echo* Ehocardiogram, *ESR* Erythrocyte sedimentation rate, *HbA1c* Hemoglobin A1C, *HIV* Human immunodeficiency viruse, *HSV* Herpes simplex virus, *Mpox* Monkeypox, *MRI* Magnetic resonance imaging, *MSM* Men Who Have Sex with Men, *NA* Not Available, *PCR* Polymerase chain reaction, *TEE* Transesophageal Echocardiography

### Baseline characteristics and data analysis

The included studies represent 13 Mpox cases with cardiac complications. Elevn cases were male patients. Five male patients have a history of having sex with men [10, 11, 13, 14, 17]. The patients who developed pericarditis or myocarditis have evidence of systemic inflammatory response in the form of fever, myalgia, and headache. Additionally, 11 patients developed a maculopapular eruption that is sometimes umbilicated [10–17]. The location of the rash was related to the site of sexual contact, including genital, anal, or oral regions. The presentation of myocarditis in the afflicted patients was usually manifested with chest pain that sometimes radiated to the left arm. The history of pox virus vaccination was either unavailable or negative in all selected patients. Despite the absence of clear vaccination history, three patients have a syphilis history [11, 16], with one patient having a history of well-controlled HIV [13]. Other sexually transmitted infections were routinely excluded from most of the patients. However, in one study, the analysis of other sexually transmitted infections was rejected due to safety considerations [13]. ECG was routinely utilized as an initial cardiac assessment tool. Different findings have been demonstrated, including sinus tachycardia, repolarization abnormality (e.g., T wave inversion), and widespread ST-segment elevation. The treatment was usually supportive because the patients presented with mild complications. However, one patient has been prescribed doses of aspirin aiming to relieve the pericarditis [12].

Real-time Polymerase Chain Reaction (PCR) targeting the virus nucleic material from the skin lesion was surely the only method to diagnose the current infection with Mpox. Various methods are used to verify the pericarditis/myocarditis diagnosis. High-sensitivity serum troponin was used to diagnose associated myocarditis in most patients. In the included cases, we found elevated levels of High-sensitivity troponins (0.165–21.20 ng/ml) [11, 17], Creatine Kinase reached (291–740 U/L) [10, 13], and N-terminal prohormone B-type natriuretic peptide (155–1258 pg/ml) [10, 11]. In addition, a serial electrocardiogram was usually used to aid the diagnosis of pericarditis. Four patients have required cardiac magnetic resonance (CMR) imaging to confirm the myocarditis [10, 13, 15, 17]. Besides, eight patients have utilized echocardiography either (transthoracic (TTE) or transesophageal) to visualize the cardiac function and pericardial fluid collection properly [10–15, 17].

Magnetic resonance imaging occasionally demonstrated the inflammation location despite transesophageal echocardiography. In T2-weighted images, areas of increased signal intensity in the basal inferior and lateral segments were found, corresponding to myocardial edema [10]. Late gadolinium enhancement sequences revealed subepicardial enhancement in the mid inferolateral segment and mid-wall enhancement in the remaining inferior and lateral segments of the LV, findings compatible with necrosis [10]. In addition, parametric mapping demonstrated a regional increase of T1 and T1 native values, indicating an abnormally expanded myocardial extracellular volume in the lateral wall. Postcontrast T1 mapping confirmed myocardial gadolinium accumulation with a nonischemic pattern in the lateral wall [10].

### Quality assessment

We have used the NIH quality assessment tool for case series to assess the quality of the included studies as previously described [9]. Three of the included studies have a good quality (> 6) [3, 11, 16], Five studies have a fair quality (from 3 to 6 points) [12–15], and one study has a poor quality (< 3) [10] as shown in Table 2.

**Table 2:** The quality of included studies using the NIH tool for case series

Author (YOP)	Q1	Q2	Q3	Q4	Q5	Q6	Q7	Q8	Q9	Overall rating
Santaliz-Ruiz et al. 2023 [17]	Y	Y	U	U	Y	Y	Y	U	Y	Fair
Brouillard et al. 2022 [15]	Y	Y	U	U	Y	Y	N	U	Y	Fair
Dumont et al. 2022 [14]	Y	Y	Y	Y	U	N	U	U	Y	Fair
Miller et al. 2022 [16]	Y	Y	Y	Y	Y	Y	Y	U	Y	Good
Pinho et al. 2022 [10]	N	N	U	U	N	N	U	U	N	Poor
Rodriguez-Nava et al. 2022 [11]	Y	Y	Y	Y	Y	Y	Y	U	Y	Good
Shaik et al. 2022 [12]	Y	Y	U	U	Y	U	Y	U	Y	Fair
Tan et al. 2022 [13]	Y	Y	U	U	Y	Y	Y	U	Y	Fair
Thornhill et al.2022 [3]	Y	Y	Y	U	Y	Y	Y	Y	Y	Good

Q1: Was the study question or objective clearly stated? Yes/No/Unclear

Q2: Was the study population clearly and fully described, including a case definition?

Q3: Were the cases consecutive?

Q4: Were the subjects comparable?

Q5: Was the intervention clearly described?

Q6: Were the outcome measures clearly defined, valid, reliable, and implemented consistently across all study participants?

Q7: Was the length of follow-up adequate?

Q8: Were the statistical methods well-described?

Q9: Were the results well-described?

*YOP* Year of publication, *Y* Yes, *N* No, *U* Unclear, *NIH* National institute of health

## Discussion

The declaration of Mpox as a public health emergency by the WHO on July 23, 2022, has raised awareness and alertness about the disease's manifestations and complications [18]. We included nine studies that reported possible cardiac complications of Mpox in which a total of 13 patients were presented [3, 10–17]. Five male patients had sex with men [10, 11, 13, 14, 17] and three male patients had unprotected sexual intercourses [11, 14] which reveal the importance of the sexual route in the disease transmission as it was reported by the CDC. Sexual contact with an infected Mpox patient is considered the main route of disease transmission during the current outbreak [19]. According to CDC, monkeypox infection is identified more in males [20].

In Mpox cases: The history began with typical Mpox symptoms such as fever, anal pain, and pustules on the face and penis a few days (four-seven days) after the last unprotected homosexual intercourse or with at-risk partners. Then, the Mpox diagnosis is confirmed by PCR. A few days later (two-seven days), patients presented with acute chest pain, elevated cardiac markers, and biological inflammatory syndrome. Typical electrocardiogram and TTE abnormalities associated with myocarditis were also identified [3, 10–17].

Few cases of cardiac involvement in Mpox infection have been reported. However, according to smallpox infection, genetically related to the Mpox virus but more aggressive, was associated with myocarditis. Furthermore, cardiac complications of smallpox vaccination have been reported since the initiation of vaccination in the 1950s in Europe, including post-vaccinal myocarditis and myopericarditis [21]. Both the live replicating smallpox vaccine ACAM2000 and the live non-replicating smallpox vaccine JYNNEOS, which are used to immunize people against the Mpox virus, have a high incidence of myocarditis but the exact mechanism remains unclear [22, 23]. On the other hand, some studies suggested two potential mechanisms of smallpox vaccine-related myopericarditis. The virus may affect the myocardium directly or by immune-mediated reaction, as evidenced by several studies that found a Th1-predominant cytokine profile associated with myocarditis after smallpox vaccination [24–26]. As *Orthopoxviruses* are closely related to the Vaccinia virus used in vaccination, it is reasonable that Mpox may also be associated with myocarditis. Extrapolating, the Mpox virus may have a tropism for myocardium tissue or cause immune-mediated injury to the heart [10].

Pericarditis is characterized by pericardial inflammation responding to various stimuli that cause an autoimmune or inflammatory response. It may result in pericardial effusion, impairing cardiac filling [27]. As most acute episodes of pericarditis are preceded by a flu-like or gastrointestinal syndrome, the etiological agents behind pericarditis are predominantly viruses [27, 28]. Myocarditis is an inflammatory myocardium disease diagnosed by established histological, immunological, and immune-histochemical criteria. It is considered a challenging diagnosis because of its heterogeneity in clinical presentation and histological forms [29].

A combination of clinical presentation and noninvasive diagnostic findings, including typical CMR abnormalities, may be used to diagnose clinically suspected myocarditis [30, 31]. The three diagnostic criteria of pericardial inflammation (chest pain, a friction rub, and diffuse ECG changes, including ST elevation) may be used to diagnose clinically suspected pericarditis [28]. The history and clinical presentation may suggest a specific etiology in patients with clinically suspected and confirmed myocarditis, but a definitive cause is often difficult to identify. As mentioned in Table 1, five cases presented without any history of potential etiology of myocarditis or pericarditis. So, Mpox infection may be the etiology of heart injuries in those patients [10, 13, 14, 17].

The management of viral myocarditis depends on the presenting symptoms, and the patient must be initially diagnosed appropriately by rolling out any other causes of the presenting symptoms [32]. Regarding our scope of cardiac complications, we will focus on managing pericarditis and myocarditis. Pericarditis can be managed by using Colchicine 1–2 mg as an initial dose that will be decreased gradually, acetylsalicylic acid, and a lower dose of nonsteroidal anti-inflammatory drugs (NSAIDs) can be used as symptomatic treatment [33, 34]. Patients who were given aspirin (1.2 g) twice daily for two weeks showed significant improvement. These patients displayed signs of recovery within eight days [12, 34]. Randomized controlled trials demonstrate colchicine's efficacy and rapid recovery in pericarditis patients [35] and significantly reduce the rate of subsequent recurrences of pericarditis in patients with multiple recurrences [36]. Patients who do not respond to colchicine and NSAIDs receive low-dose steroids as a second line of treatment [34].

Our included myocarditis cases received supportive care, exercise restriction, and the initiation of ibuprofen and colchicine if the case was combined with pericarditis (myopericarditis). However, they did not receive any directed therapy for myocarditis. The patients had a full clinical recovery in one to two weeks. They were discharged with normal cardiac enzymes and sustained electric and hemodynamic stability [10, 11, 13, 15]. Three myocarditis cases received cardioprotective treatment (Bisoprolol and Ramipril) [10, 11, 13]. This treatment resulted in no recurrence of pain, and high-sensitivity troponin T decreased [14]. According to Miller et al. 2022 (newly included study), four cases received Tecovirimat as an antiviral drug for the 14-day duration with supportive treatment [11, 14–16], as recommended by the CDC guidelines [37]. Because those cases evolved rapidly, one of the cases left the hospital early before the assessment of the effect of Tecovirimat on myocardial inflammation without a follow-up CMR [15].

Early implementation of supportive care and pain control is recommended. The effectiveness of this measure is based on the patient's immune system. Usually, these treatments are sufficient for immunocompetent patients. Nevertheless, the illness outcome is determined by several factors, including the patient's initial health condition, existing diseases, immunization record, and co-morbidity. Thus, supportive care and pain control may not be enough for some people with weakened immune systems, and Tecovirimat should be considered [14, 16].

Tecovirimat is a drug that suppresses the activity of the VP37 envelope-wrapping protein of *Orthopoxviruses*, thus hindering the production of enveloped virions and diminishing the amount of viral DNA in the blood of a non-human primate organism [37]. While there is no data available on the effectiveness of Tecovirimat for treating people with Mpox, experiments conducted with a variety of animal species have yielded positive results regarding its ability to lessen the likelihood of death from Orthopoxviruses if it is taken early in the disease [38]. Furthermore, FDA declared that the indiscriminate usage of Tecovirimat might induce viral resistance making Tecovirimat ineffective for orthodox-infected patients [37]. Alternate treatment options have a less safety profile than Tecovirimat. So, when Tecovirimat is needed, it is important to be aware of the lack of evidence on its effectiveness for Mpox patients and the possibility of viral resistance. Tecovirimat is accessible from the Strategic National Stockpile of the United States and should be given out under the rigid guidelines of the CDC Institutional Review Board. As per the CDC's advice, the medication should be considered for patients with intense illness, those in danger of developing a serious disease, and those whose body parts are at risk of being affected by the virus (such as the genitals) [37].

## Strengths and limitations

The small number of the reported Mpox cases with cardiac complications limits the study. All the available studies are case reports or case series. Additionally, we had to include some studies despite missing data due to the lack of these cases. Some of the included cases have a history of HIV and syphilis which may impact the patients' immunity. These limitations reduce the evidence quality of this study.

The strong points of this study: this systematic review is novel to aggregate cardiac complications in monkeypox. Also, this study illustrates the importance to diagnose cardiac complications early which can improve patient outcomes.

## Conclusion and recommendations

This study suggests the possibility of cardiac complications in Mpox patients. So, we advise doing cardiovascular investigations for the Mpox patients with suspected cardiovascular affectionNo mortality was associated with the documented mild myocarditis and/or pericarditis sequelae. Further research is needed to identify the pathological mechanism underlying Mpox-associated heart injury. Also, we recommend conducting clinical trials to evaluate Tecovirimat's efficacy and safety in managing Mpox patients presented with cardiac complications.

## Data Availability

Data will be provided upon request from Reem Sayad (reem.17289806@med.aun.edu.eg).

## Abbreviations

CMR: Cardiac magnetic resonance
CDC: Center for Disease Control and Prevention
ECG: Electrocardiography
MSM: Men Who Have Sex with Men
Mpox: Monkeypox
NSAIDs: Nonsteroidal anti-inflammatory drugs
PCR: Polymerase Chain Reaction
TTE: Transthoracic echocardiography
WHO: World Health Organization
